# Reliability Modeling Method for Constant Stress Accelerated Degradation Based on the Generalized Wiener Process

**DOI:** 10.3390/e27121197

**Published:** 2025-11-26

**Authors:** Shanshan Li, Zaizai Yan, Junmei Jia

**Affiliations:** College of Science, Inner Mongolia University of Technology, Hohhot 010051, China; lishanshan501@163.com (S.L.);

**Keywords:** generalized Wiener process, accelerated degradation model, random effects, maximum likelihood, expectation maximization

## Abstract

This paper aims to improve the accuracy of reliability estimates and the failure time prediction for products exhibiting nonlinear degradation behavior under constant-stress accelerated degradation test (CSADT). To achieve this, a novel degradation model and a life prediction method are proposed, which are based on a generalized Wiener process. Some models assume that the drift coefficients are related to accelerated stress. However, in certain applications, the diffusion coefficients are also affected by accelerated stress. The relationship between the drift parameter and accelerated stress variables can be derived by the accelerated model, and so is the relationship between the diffusion parameter and stress variables based on the principle of invariance of the acceleration factor. To account for individual variability among products, random effects are introduced. Model parameters are estimated using a combination of maximum likelihood estimation (MLE) and the expectation-maximization (EM) algorithm. Furthermore, the probability density function (PDF) of the remaining useful life under normal stress conditions is derived using the law of total probability. The effectiveness and applicability of the proposed approach are validated using simulated constant stress accelerated degradation data and stress relaxation data. The results demonstrate that the model not only fits the degradation process well but also modestly improves the accuracy of the failure time prediction, providing valuable guidance for engineering maintenance and reliability management.

## 1. Introduction

Accelerated degradation test (ADT) is widely employed in the reliability assessment of high-reliability and long-life products [1]. Since many such products degrade slowly under normal stress conditions, ADT can significantly reduce testing time, lower costs, and improve efficiency while ensuring consistency of the failure mechanisms [2]. Addressing reliability problems characterized by diverse uncertainties, Wang et al. [3] developed a novel model and method for hybrid reliability optimization design. Their study fits within the broader field of uncertainty quantification, in which mathematical modeling approaches have been comprehensively reviewed, including theoretical foundations and recent developments in probabilistic, non-probabilistic, and hybrid frameworks [4]. Due to the time-varying uncertainty during the degradation process, Son et al. [5] argued that stochastic processes provide more suitable descriptions of the product degradation patterns. The Wiener process, a well-established stochastic model, is widely used in degradation analysis due to its stationary nature, desirable mathematical properties, and clear physical interpretability [6]. Whitmore and Schenkelberg [7] introduced a nonlinear Wiener process based on time-scale transformation, successfully predicting the service life of self-regulating heating cables. However, in some cases, time-scale transformation cannot linearize the nonlinear degradation process, making the direct construction of nonlinear drift degradation models a more suitable approach. Si et al. [8] proposed a nonlinear Wiener process model, deriving an approximate analytical expression for the probability distribution of the remaining lifetime. Furthermore, Wang et al. [9] developed a generalized Wiener process model that incorporates both nonlinear degradation characteristics and temporal uncertainty, where the temporal uncertainty is represented by a non-standard Brownian motion. This model is highly generalizable and encompasses several commonly used Wiener process models.

Accelerated degradation tests are primarily categorized into the constant-stress accelerated degradation test (CSADT) and step-stress accelerated degradation test (SSADT), depending on how stress is applied [10]. In CSADT, test samples are divided into groups, each subjected to a different stress level, which remains constant throughout the test. Due to its simplicity and ease of implementation, CSADT is a widely adopted approach in ADT studies [11]. Duan et al. [12] proposed a reliability analysis approach for degradation data based on a time-scale transformation of the nonlinear Wiener process, incorporating mixed random effects under CSADT conditions.

To ensure accurate reliability analysis, it is essential to account for individual heterogeneity in the degradation process [13]. In ADT, even among similar products, degradation performance often varies across samples and exhibits a certain pattern, commonly referred to as random effects. Numerous researchers have explored the influence of random effects in accelerated degradation scenarios. Wang et al. [14] introduced the principle of invariant acceleration factors and examined the impact of random effects in CSADT by employing a Wiener process with time-scale transformation. Tang et al. [15] incorporated normally distributed random effects into the degradation framework, utilizing a time-scale transformed Wiener process to perform nonlinear degradation characterization and evaluate the reliability of light-emitting diodes (LEDs) under CSADT conditions. Liu et al. [16] explored generalized Wiener process-based accelerated degradation models for both CSADT and SSADT, incorporating both sample variability and multiple accelerating stress factors. Li et al. [17] developed a CSADT model that accounts for random effects, stress conditions, and measurement errors.

The generalized Wiener process has been extensively applied in degradation modeling due to its strong generality, as it encompasses a wide range of common Wiener process models. Under accelerated conditions, the degradation process of a product exhibits significant stochastic behavior. Therefore, it is appropriate to adopt the generalized Wiener process to characterize the accelerated degradation trajectory. Accelerated stress influences the drift coefficient and the diffusion coefficient; the proposed degradation model is constructed based on the principle of invariance of the acceleration factor. This approach not only enhances the model’s applicability but also enables a more accurate description of the product’s degradation path, thereby improving the precision of the failure time prediction. In this paper, the fluctuation of performance degradation within individual products is modeled using a generalized Wiener process, which not only captures the effects of accelerated stress on both the drift and diffusion coefficients but also incorporates random effects to reflect variability among products. Wan et al. [18] combined the MLE method and the EM algorithm for parameter estimation in nonlinear Wiener processes. Based on this conclusion, the maximum likelihood estimation (MLE) method and the expectation-maximization (EM) algorithm are employed to estimate the model parameters.

The structure of this paper is organized as follows: Section 2 presents the generalized Wiener process model, in which both the drift and diffusion coefficients are influenced by accelerated stress. Section 3 discusses the parameter estimation methods and details the implementations of the MLE and EM algorithms. Section 4 compares the efficiency of four models using a numerically simulated example and stress relaxation data, demonstrating the superiority of the proposed model. Finally, Section 5 provides a summary of the study and offers an outlook on future research.

## 2. Modeling of Constant Stress Accelerated Degradation

### 2.1. Generalized Wiener Process

Assume that the degradation process of a product follows a generalized Wiener process. Let $X\left(t\right)$ denote the degradation amount at time t. The generalized Wiener process degradation model can be expressed as follows:

(1) $$X\left(t\right)=X\left(0\right)+\mu \Lambda \left(t;\theta \right)+\sigma_{B}B\left(\tau \left(t;\gamma \right)\right)$$

where $X\left(0\right)=0$ is the initial degradation state, Generally speaking, $\Lambda \left(t;\theta \right)$ and $\tau \left(t;\gamma \right)$ are monotonically non-decreasing nonlinear functions and continuous of time t, θ and γ are parameters of the time-scale function, μ is the drift coefficient, $\sigma_{B}$ is the diffusion coefficient, $B\left(\tau \left(t;\gamma \right)\right)$ is a nonstandard Brownian motion if $\tau \left(t;\gamma \right)\neq t$, and is employed to characterize the temporal uncertainty in the degradation process.

**Lemma** **1** ([9])**.** *Let* D *be the degradation failure threshold, and let* T *denote the first passage time (FHT) at which the degradation process reaches the failure threshold*

$T=\inf\left\{t:X\left(t\right)\geq D\right\}$*. According to the definition of the FHT, the conditional probability density function (PDF) of failure time, given* μ *, is approximated as follows:*

$$f_{1}\left(t|\mu \right)≃\frac{1}{A_{1}}g_{1}\left(t|\mu \right)$$

(2) $$g_{1}\left(t|\mu \right)≃\frac{1}{\sqrt{2\pi \tau \left(t;\gamma \right)}}\left[\frac{Q\left(t\right)}{\tau \left(t;\gamma \right)}+\frac{\mu h\left(\tau \left(t;\gamma \right);\theta \right)}{\sigma_{B}}\right]\exp\left[-\frac{Q^{2}\left(t\right)}{2\tau \left(t;\gamma \right)}\right]\frac{d\tau \left(t;\gamma \right)}{dt}$$

*where* $h\left(\tau \left(t;\gamma \right);\theta \right)=\frac{d\Lambda \left(\tau^{-1}\left(s;\gamma \right);\theta \right)}{ds}{|}_{s=\tau \left(t;\gamma \right)}$, $A_{1}=\int_{0}^{\infty }g_{1}\left(t|\mu \right)dt$, $Q\left(t\right)=\frac{D-\mu \Lambda \left(t;\theta \right)}{\sigma_{B}}$.

**Case 1**. When γ=1, $X(t)=\mu \Lambda \left(t;\theta \right)+\sigma_{B}B\left(t\right)$, the degradation process follows the nonlinear Wiener process model proposed by Si et al. [8], and the conditional PDF for failure time is given by Equation (3).

(3) $$f_{2}\left(t|\mu \right)=\frac{1}{\sqrt{2\pi \sigma_{B}^{2}t^{3}}}\left[D-\mu \left(\Lambda \left(t;\theta \right)-t\frac{d\Lambda \left(t;\theta \right)}{dt}\right)\right]\exp\left[-\frac{{\left(D-\mu \Lambda \left(t;\theta \right)\right)}^{2}}{2\sigma_{B}^{2}t}\right]$$

**Case 2**. When θ=γ, $X\left(t\right)=\mu \Lambda \left(t;\theta \right)+\sigma_{B}B\left(\Lambda \left(t;\theta \right)\right)$, the degradation is modeled as a time-scale transformed Wiener process [7]. Given μ, the conditional PDF of a product’s failure time is expressed as Equation (4).

(4) $$f_{3}\left(t|\mu \right)=\frac{D}{\sqrt{2\pi \sigma_{B}^{2}\Lambda^{3}\left(t;\theta \right)}}\exp\left[-\frac{{\left(D-\mu \Lambda \left(t;\theta \right)\right)}^{2}}{2\sigma_{B}^{2}\Lambda \left(t;\theta \right)}\right]\frac{d\Lambda \left(t;\theta \right)}{\mathrm{dt}}$$

### 2.2. Generalized Wiener Process Model with Accelerated Stress and Random Effects

It is assumed that there are stress levels m, $S_{0}<\cdots <S_{m}$, where $S_{0}$ represents the normal use stress and $S_{m}$ denotes the highest allowable stress. The acceleration models commonly employed include the Arrhenius model, the Power law model, and the Exponential model. When the accelerating stress is electrical, the acceleration model follows the Power law model. For convenience, normalized the stress as $s_{i}=\frac{\ln S_{0}-\ln S_{i}}{\ln S_{0}-\ln S_{m}}$, $s_{i}\in \left[0,1\right]$, $\eta_{i}\left(b\right)=\exp(bs_{i})$, i=1,2,⋯,m, where b is an unknown parameter. In the same way, when the accelerating stress is temperature, the acceleration model follows the Arrhenius model [19]. Normalized the stress level as $s_{i}=\frac{1/\left(S_{0}+273.15\right)-1/\left(S_{i}+273.15\right)}{1/\left(S_{0}+273.15\right)-1/\left(S_{m}+273.15\right)}$, where $S_{i}$ denotes the temperature in degrees Celsius (°C), while $S_{i}+273.15$ represents the corresponding value in Kelvin (K), i=1,2,⋯,m. Based on the nonlinear Wiener process of time scale transformation, the acceleration factor $AF_{k,h}$ of stress $s_{k}$ relative to stress $s_{h}$ is constant. The literature [20] deduces the relationship: $AF_{k,h}^{c}=\frac{\mu_{k}}{\mu_{h}}=\frac{{\left(\sigma_{B}^{2}\right)}_{k}}{{\left(\sigma_{B}^{2}\right)}_{h}}$, $\mu_{k}$, $\mu_{h}$ are the drift coefficients under stress $s_{k}$, $s_{h}$, ${\left(\sigma_{B}^{2}\right)}_{k}$, ${\left(\sigma_{B}^{2}\right)}_{h}$ are the diffusion coefficients under stress $s_{k}$, $s_{h}$, and the time function under each stress is $\Lambda \left(t;c\right)=t^{c}$. Based on the principle of invariance of the acceleration factor [20], it is deduced that the ratio of the drift coefficient to the diffusion coefficient in the nonlinear Wiener process is constant, and both are related to the accelerating stress, rather than assuming that the diffusion parameter is constant and independent of stress. The nonlinear Wiener process based on time-scale transformation is a special form of the generalized Wiener process. By using the proof-by-contradiction method, it can be concluded that there is a correlation between the diffusion coefficient and the accelerating stress in the accelerated degradation model based on the generalized Wiener process [21]. The model proposed by Zheng et al. [1] in the step-stress accelerated degradation test considers both the drift coefficient and the diffusion coefficient, which are related to the accelerated stress. When using the general nonlinear Wiener process for accelerated degradation data analysis, it is usually assumed that the diffusion coefficient is a constant [22]. If the drift coefficient of the $j^{th}$ product under stress $s_{i}$ is denoted as $\mu_{ij}=a_{ij}\eta_{i}\left(b\right)$, where $a_{ij}∼N\left(\mu_{a},\sigma_{a}^{2}\right)$ represents the variability among product samples [23], the corresponding diffusion coefficient is $q\eta_{i}\left(b\right)$, assuming the diffusion coefficient does not account for individual product variability and that q is a fixed-effects parameter. The generalized Wiener process is given by $M_{1}$:$X(t)=\mu_{ij}\Lambda \left(t;\theta \right)+q\eta_{i}\left(b\right)B\left(\tau \left(t;\gamma \right)\right)$, where the drift coefficient $a_{ij}∼N\left(\mu_{a},\sigma_{a}^{2}\right)$, i=1,2,⋯,m, $j=1,2,\cdots ,n_{i}$. To facilitate the derivation of the PDF for the failure time influenced by the random variable $a_{ij}$, the following lemma is presented.

**Lemma** **2** ([24])**.** *If* $Z∼N\left(\mu ,\sigma^{2}\right)$ *,* $w_{1}$ *,* $w_{2}$ *,* A *,* B∈R *and* $C\in R^{+}$ *hold, then*

$$E_{Z}\left\{\left(w_{1}-AZ\right)\exp\left[-\frac{{\left(w_{2}-BZ\right)}^{2}}{2C}\right]\right\}$$

(5) $$=\sqrt{\frac{C}{B^{2}\sigma^{2}+C}}\left(w_{1}-A\frac{Bw_{2}\sigma^{2}+\mu C}{B^{2}\sigma^{2}+C}\right)\exp\left[-\frac{{\left(w_{2}-B\mu \right)}^{2}}{2\left(B^{2}\sigma^{2}+C\right)}\right]$$

**Theorem** **1.** *When the random variable* $a_{ij}∼N\left(\mu_{a},\sigma_{a}^{2}\right)$ *, the PDF of failure time under stress level* $s_{i}$ *can be approximated as follows:*

$$f_{1}\left(t|s_{i}\right)≅\frac{1}{A_{M1}}g_{1}\left(t|s_{i}\right)$$

$$g_{1}\left(t|s_{i}\right)≃\frac{1}{\tau \left(t;\gamma \right)\sqrt{2\pi \left(\sigma_{a}^{2}\eta_{i}\left(2b\right)\Lambda^{2}\left(t;\theta \right)+q\eta_{i}\left(b\right)\tau \left(t;\gamma \right)\right)}}\frac{d\tau \left(t;\gamma \right)}{dt}$$

$$\left\{D-[\Lambda \left(t;\theta \right)-h\left(\tau \left(t;\gamma \right);\theta \right)\tau \left(t;\gamma \right)]\frac{\sigma_{a}^{2}D\eta_{i}\left(b\right)\Lambda \left(t;\theta \right)+\mu_{a}q\eta_{i}\left(b\right)\tau \left(t;\gamma \right)}{\sigma_{a}^{2}\eta_{i}\left(b\right)\Lambda^{2}\left(t;\theta \right)+q\tau \left(t;\gamma \right)}\right\}$$

(6) $$\exp\left[-\frac{{\left(D-\mu_{a}\eta_{i}\left(b\right)\Lambda \left(t;\theta \right)\right)}^{2}}{2\left(\sigma_{a}^{2}\eta_{i}\left(2b\right)\Lambda^{2}\left(t;\theta \right)+q\eta_{i}\left(b\right)\tau \left(t;\gamma \right)\right)}\right]$$

*where* $h\left(\tau \left(t;\gamma \right);\theta \right)=\frac{d\Lambda \left(\tau^{-1}\left(u;\gamma \right);\theta \right)}{du}{|}_{u=\tau \left(t;\gamma \right)}$, $A_{M1}=\int_{0}^{\infty }g_{1}\left(t|s_{i}\right)dt$*. The corresponding reliability function is denoted as* $R_{1}\left(t|s_{i}\right)=1-\int_{0}^{t}f_{1}\left(u|s_{i}\right)du$*.*

**Proof.**  $f_{1}\left(t|s_{i}\right)=\int_{-\infty }^{+\infty }f_{1}\left(t|a_{ij},s_{i}\right)p(a_{ij})da_{ij}$, $p(a_{ij})$ represents the PDF of $a_{ij}$.

$$f_{1}\left(t|a_{ij},s_{i}\right)≃\frac{1}{\sqrt{2\pi q\eta_{i}\left(b\right)\tau \left(t;\gamma \right)}}\frac{d\tau \left(t;\gamma \right)}{dt}\exp\left[-\frac{{\left(D-\mu_{ij}\eta_{i}\left(b\right)\Lambda \left(t;\theta \right)\right)}^{2}}{2q\eta_{i}\left(b\right)\tau \left(t;\gamma \right)}\right]$$

$$\left[\frac{D-\mu_{ij}\eta_{i}\left(b\right)\Lambda \left(t;\theta \right)}{\tau \left(t;\gamma \right)}+\mu_{ij}\eta_{i}\left(b\right)h\left(\tau \left(t;\gamma \right);\theta \right)\right]$$

$$f_{1}\left(t|a_{ij},s_{i}\right)≃\frac{1}{\tau \left(t;\gamma \right)\sqrt{2\pi q\eta_{i}\left(b\right)\tau \left(t;\gamma \right)}}\frac{d\tau \left(t;\gamma \right)}{dt}\exp\left[-\frac{{\left(D-\mu_{ij}\eta_{i}\left(b\right)\Lambda \left(t;\theta \right)\right)}^{2}}{2q\eta_{i}\left(b\right)\tau \left(t;\gamma \right)}\right]$$

$$\left[D-\mu_{ij}\eta_{i}\left(b\right)\left(\Lambda \left(t;\theta \right)-\tau \left(t;\gamma \right)h\left(\tau \left(t;\gamma \right);\theta \right)\right)\right]$$

$$w_{1}=D,\;w_{2}=D,\;A=\eta_{i}\left(b\right)\left[\Lambda \left(t;\theta \right)-\tau \left(t;\gamma \right)h\left(\tau \left(t;\gamma \right);\theta \right)\right],\;C=q\eta_{i}\left(b\right)\tau \left(t;\gamma \right),$$

$B=\eta_{i}\left(b\right)\Lambda \left(t;\theta \right)$, and according to Lemma 2, Equation (6) follows directly.

When γ=1, the model is denoted as $M_{2}$: $X(t)=a_{ij}\eta_{i}\left(b\right)\Lambda \left(t;\theta \right)+q\eta_{i}\left(b\right)B\left(t\right)$, and the PDF of failure time is given by

$$f_{2}\left(t|s_{i}\right)≅\frac{1}{A_{M2}}g_{2}\left(t|s_{i}\right)$$

$$g_{2}\left(t|s_{i}\right)≃\frac{1}{t\sqrt{2\pi \left(\sigma_{a}^{2}\eta_{i}\left(2b\right)\Lambda^{2}\left(t;\theta \right)+q\eta_{i}\left(b\right)t\right)}}\exp\left[-\frac{{\left(D-\mu_{a}\eta_{i}\left(b\right)\Lambda \left(t;\theta \right)\right)}^{2}}{2\left(\sigma_{a}^{2}\eta_{i}\left(2b\right)\Lambda^{2}\left(t;\theta \right)+q\eta_{i}\left(b\right)t\right)}\right]$$

(7) $$\left\{D-[\Lambda \left(t;\theta \right)-t\frac{d\Lambda \left(t;\theta \right)}{\mathrm{dt}}]\frac{\sigma_{a}^{2}D\eta_{i}\left(b\right)\Lambda \left(t;\theta \right)+\mu_{a}q\eta_{i}\left(b\right)t}{\sigma_{a}^{2}\eta_{i}\left(b\right)\Lambda^{2}\left(t;\theta \right)+qt}\right\}$$

where $A_{M2}=\int_{0}^{\infty }g_{2}\left(t|s_{i}\right)dt$. The corresponding reliability function is expressed as $R_{2}\left(t|s_{i}\right)=1-\int_{0}^{t}f_{2}\left(u|s_{i}\right)du$.

When θ=γ, the model is denoted as $M_{3}$: $X(t)=\mu_{ij}\Lambda \left(t;\theta \right)+q\eta_{i}\left(b\right)B\left(\Lambda \left(t;\theta \right)\right)$, the PDF of failure time is given by Equation (8).

$$f_{3}\left(t|s_{i}\right)≃\frac{D}{\Lambda \left(t;\theta \right)\sqrt{2\pi \left(\sigma_{a}^{2}\eta_{i}\left(2b\right)\Lambda^{2}\left(t;\theta \right)+q\eta_{i}\left(b\right)\Lambda \left(t;\theta \right)\right)}}\frac{d\Lambda \left(t;\theta \right)}{dt}$$

(8) $$\exp\left[-\frac{{\left(D-\mu_{a}\eta_{i}\left(b\right)\Lambda \left(t;\theta \right)\right)}^{2}}{2\left(\sigma_{a}^{2}\eta_{i}\left(2b\right)\Lambda^{2}\left(t;\theta \right)+q\eta_{i}\left(b\right)\Lambda \left(t;\theta \right)\right)}\right]$$

and the corresponding reliability function is expressed as Equation (9).

$$R_{3}\left(t|s_{i}\right)=\Phi \left\{\frac{D-\mu_{a}\eta_{i}\left(b\right)\Lambda \left(t;\theta \right)}{\sqrt{\sigma_{a}^{2}\eta_{i}\left(2b\right)\Lambda^{2}\left(t;\theta \right)+q\eta_{i}\left(b\right)\Lambda \left(t;\theta \right)}}\right\}-\exp\left(\frac{2D\left(\sigma_{a}^{2}D+\mu_{a}q\right)}{q^{2}}\right)$$

(9) $$\Phi \left\{-\frac{2D\sigma_{a}^{2}\eta_{i}\left(b\right)\Lambda \left(t;\theta \right)+q\left[\mu_{a}\eta_{i}\left(b\right)\Lambda \left(t;\theta \right)+D\right]}{q\sqrt{\sigma_{a}^{2}\eta_{i}\left(2b\right)\Lambda^{2}\left(t;\theta \right)+q\eta_{i}\left(b\right)\Lambda \left(t;\theta \right)}}\right\}$$

If individual variability is not considered, the model is expressed as $M_{4}$: $X(t)=a\eta_{i}\left(b\right)\Lambda \left(t;\theta \right)+\sigma_{B}B\left(\tau \left(t;\gamma \right)\right)$, where a is a fixed parameter. By substituting the drift coefficient $\mu =a\eta_{i}\left(b\right)$ into Equation (2), the PDF of failure time is given by Equation (10).

(10) $$f_{4}\left(t|s_{i}\right)=\frac{1}{\sqrt{2\pi q\eta_{i}\left(b\right)\Lambda^{3}\left(t;\theta \right)}}\exp\left[-\frac{{\left(D-a\eta_{i}\left(b\right)\Lambda \left(t;\theta \right)\right)}^{2}}{2q\eta_{i}\left(b\right)\Lambda \left(t;\theta \right)}\right]\frac{d\Lambda \left(t;\theta \right)}{dt}$$

and the corresponding reliability function is Equation (11).

(11) $$R_{4}\left(t|s_{i}\right)=\Phi \left[\frac{D-a\eta_{i}\left(b\right)\Lambda \left(t;\theta \right)}{\sqrt{q\eta_{i}\left(b\right)\Lambda \left(t;\theta \right)}}\right]-\exp\left(\frac{2aD}{q}\right)\Phi \left[-\frac{D+a\eta_{i}\left(b\right)\Lambda \left(t;\theta \right)}{\sqrt{q\eta_{i}\left(b\right)\Lambda \left(t;\theta \right)}}\right].$$

□

## 3. Parameter Estimation

The unknown parameters in the CSADT models are $\Theta =\left\{b,\theta ,\gamma ,\mu_{a},\sigma_{a}^{2},q\right\}$. Let the increment of the $k^{th}$ measurement over the ${\left(k-1\right)}^{th}$ measurement for the $j^{th}$ sample at the normalized stress level $s_{i}$ be denoted as $\Delta x_{i,k}^{j}∼N\left(a_{ij}\eta_{i}\left(b\right)\Delta \Lambda \left(t_{i,k}^{j},\theta \right),q\eta_{i}\left(b\right)\Delta \tau \left(t_{i,k}^{j},\gamma \right)\right)$,

$$\mathrm{where}\;\Delta x_{i,k}^{j}=X\left(t_{i,k}^{j}\right)-X\left(t_{i,k-1}^{j}\right),\;i=1,2,\cdots ,m,\;j=1,2,\cdots ,n_{i},\;k=1,2,\cdots ,p_{i},$$

$\Delta \Lambda \left(t_{i,k}^{j},\theta \right)={\left(t_{i,k}^{j}\right)}^{\theta }-{\left(t_{i,(k-1)}^{j}\right)}^{\theta }$ and $\Delta \tau \left(t_{i,k}^{j},\gamma \right)={\left(t_{i,k}^{j}\right)}^{\gamma }-{\left(t_{i,(k-1)}^{j}\right)}^{\gamma }$. Normally, the average degradation amount of a product is proportional to time raised to a power b [25], as seen in material aging [26] and crack growth [27]. Therefore, $\Lambda \left(t;\theta \right)=t^{\theta }$ and $\tau \left(t;\gamma \right)=t^{\gamma }$ are assumed. However, in practical applications, forms $\Lambda \left(t;\theta \right)$ and $\tau \left(t;\gamma \right)$ can also be adopted depending on the product’s degradation trajectory. The most appropriate form is then selected according to the Akaike Information Criterion (AIC).

The corresponding likelihood function is expressed as Equation (12).

(12) $$L\left(\Theta_{1}\right)=\prod_{i=1}^{m}\prod_{j=1}^{n_{i}}\prod_{k=1}^{p_{i}}\frac{1}{\sqrt{2\pi q\eta_{i}\left(b\right)\Delta \tau \left(t_{i,k}^{j},\gamma \right)}}\exp\left[-\frac{{\left(\Delta x_{i,k}^{j}-a_{ij}\eta_{i}\left(b\right)\Delta \Lambda \left(t_{i,k}^{j},\theta \right)\right)}^{2}}{2q\eta_{i}\left(b\right)\Delta \tau \left(t_{i,k}^{j},\gamma \right)}\right]$$

The log-likelihood function can be deduced as

$$\ln L\left(\Theta_{1}\right)=-\frac{1}{2}\sum_{i=1}^{m}n_{i}p_{i}\left[\ln\left(2\pi q\eta_{i}\left(b\right)\right)\right]-\frac{1}{2}\sum_{i=1}^{m}\sum_{j=1}^{n_{i}}\sum_{k=1}^{p_{i}}\Delta \tau \left(t_{i,k}^{j},\gamma \right)$$

(13) $$-\frac{1}{2}\sum_{i=1}^{m}\sum_{j=1}^{n_{i}}\sum_{k=1}^{p_{i}}\frac{{\left(\Delta x_{i,k}^{j}-a_{ij}\eta_{i}\left(b\right)\Delta \Lambda \left(t_{i,k}^{j},\theta \right)\right)}^{2}}{q\eta_{i}\left(b\right)\Delta \tau \left(t_{i,k}^{j},\gamma \right)}$$

Let $\Theta_{1}=\left\{a_{ij},q,\theta ,\gamma ,b\right\}$, $j=1,2,\cdots ,n_{i}$ denote the parameter set. Taking the partial derivatives of Equation (13) with respect to parameters $a_{ij}$, q and setting them to zero, the following results are deduced

(14) $${\hat{a}}_{ij}=\frac{\sum_{k=1}^{p_{i}}\Delta x_{i,k}^{j}\Delta \Lambda \left(t_{i,k}^{j},\theta \right)/\Delta \tau \left(t_{i,k}^{j},\gamma \right)}{\eta_{i}\left(b\right)\sum_{k=1}^{p_{i}}\Delta \Lambda^{2}\left(t_{i,k}^{j},\theta \right)/\Delta \tau \left(t_{i,k}^{j},\gamma \right)}$$

(15) $$\hat{q}=\frac{1}{\sum_{i=1}^{m}n_{i}p_{i}}\sum_{i=1}^{m}\sum_{j=1}^{n_{i}}\sum_{k=1}^{p_{i}}\frac{{\left(\Delta x_{i,k}^{j}-a_{ij}\eta_{i}\left(b\right)\Delta \Lambda \left(t_{i,k}^{j},\theta \right)\right)}^{2}}{\eta_{i}\left(b\right)\Delta \tau \left(t_{i,k}^{j},\gamma \right)}$$

By substituting Equations (14) and (15) into Equation (13), the profile log-likelihood function is simplified as Equation (16).

(16) $$\ln L\left(\Theta_{1}\right)=-\frac{1}{2}\sum_{i=1}^{m}n_{i}p_{i}\left[\ln\left(2\pi \hat{q}\eta_{i}\left(b\right)\right)+1\right]-\frac{1}{2}\sum_{i=1}^{m}\sum_{j=1}^{n_{i}}\sum_{k=1}^{p_{i}}\Delta \tau \left(t_{i,k}^{j},\gamma \right)$$

The fminsearch algorithm in MATLAB R2024a [28] is employed to estimate the parameters θ, γ and b, while the estimates for $a_{ij}$ and q are obtained simultaneously. Since $a_{ij}$ is a latent variable, solving for parameters $\mu_{a}$, $\sigma_{a}^{2}$ directly using the MLE method is challenging. The EM algorithm, an effective parameter estimation technique for handling latent variables, is employed to estimate parameters $\mu_{a}$, $\sigma_{a}^{2}$ and q. The EM algorithm, proposed by Dempster [29] in 1977, involves two steps: the first step computes the expectation of the latent variable, and the second step maximizes the likelihood function. The computational procedures for the E and M steps are outlined below.

**E-Step:** Calculate the mathematical expectation involving $a_{ij}$ and $a_{ij}^{2}$. The random variable $a_{ij}$ follows a normal distribution, $a_{ij}|\left(X_{i}^{j},\Theta_{2}\right)$ represents the probability distribution of $a_{ij}$ conditional on $X_{i}^{j}$ and $\Theta_{2}$, where $X_{i}^{j}$ is the degradation data of the $j^{th}$ product under stress $s_{i}$, $\Theta_{2}=\left\{\mu_{a},\sigma_{a}^{2},q\right\}$. ${\hat{\Theta }}_{2e}=\left\{{\left({\hat{\mu }}_{a}\right)}_{e},{\left({\hat{\sigma }}_{a}^{2}\right)}_{e},{\hat{q}}_{e}\right\}$ are the parameter estimates obtained from the $e^{th}$ iteration. The corresponding equations are

(17) $$a_{ij}|X_{i}^{j},{\hat{\Theta }}_{2e}\sim N\left(E\left(a_{ij}|X_{i}^{j},{\hat{\Theta }}_{2e}\right),\mathrm{var}\left(a_{ij}|X_{i}^{j},{\hat{\Theta }}_{2e}\right)\right)$$

(18) $$E\left(a_{ij}|X_{i}^{j},{\hat{\Theta }}_{2e}\right)=\frac{{\left({\hat{\sigma }}_{a}^{2}\right)}_{e}\sum_{k=1}^{p_{i}}\Delta x_{i,k}^{j}\Delta \Lambda \left(t_{i,k}^{j},\theta \right)/\Delta \tau \left(t_{i,k}^{j},\gamma \right)+{\hat{q}}_{e}{\left({\hat{\mu }}_{a}\right)}_{e}}{{\left({\hat{\sigma }}_{a}^{2}\right)}_{e}\eta_{i}\left(b\right)\sum_{k=1}^{p_{i}}\Delta \Lambda^{2}\left(t_{i,k}^{j},\theta \right)/\Delta \tau \left(t_{i,k}^{j},\gamma \right)+{\hat{q}}_{e}}$$

(19) $$Var\left(a_{ij}|X_{i}^{j},{\hat{\Theta }}_{2e}\right)=\frac{{\left({\hat{\sigma }}_{a}^{2}\right)}_{e}{\hat{q}}_{e}}{{\left({\hat{\sigma }}_{a}^{2}\right)}_{e}\eta_{i}\left(b\right)\sum_{k=1}^{p_{i}}\Delta \Lambda^{2}\left(t_{i,k}^{j},\theta \right)/\Delta \tau \left(t_{i,k}^{j},\gamma \right)+{\hat{q}}_{e}}$$

The expectation of the complete log-likelihood function is expressed as:

$$\ln L(\Theta_{1}|X_{i}^{j},{\hat{\Theta }}_{2e})=-\frac{1}{2}\sum_{i=1}^{m}n_{i}p_{i}\left[\ln(2\pi q\eta_{i}\left(b\right))\right]-\frac{1}{2}\sum_{i=1}^{m}\sum_{j=1}^{n_{i}}\sum_{k=1}^{p_{i}}\Delta \tau \left(t_{i,k}^{j},\gamma \right)-$$

$$\frac{1}{2q}\sum_{i=1}^{m}\sum_{j=1}^{n_{i}}\sum_{k=1}^{p_{i}}\frac{{\left(\Delta x_{i,k}^{j}-E\left(a_{ij}|X_{i}^{j},{\hat{\Theta }}_{2e}\right)\eta_{i}\left(b\right)\Delta \Lambda \left(t_{i,k}^{j},\theta \right)\right)}^{2}+Var\left(a_{ij}|X_{i}^{j},{\hat{\Theta }}_{2e}\right)\eta_{i}\left(2b\right)\Delta \Lambda^{2}\left(t_{i,k}^{j},\theta \right)}{\eta_{i}\left(b\right)\Delta \tau \left(t_{i,k}^{j},\gamma \right)}-$$

(20) $$\frac{1}{2}\sum_{i=1}^{m}n_{i}\ln(2\pi \sigma_{a}^{2})-\frac{1}{2\sigma_{a}^{2}}\sum_{i=1}^{m}\sum_{j=1}^{n_{i}}\left\{{\left[E\left(a_{ij}|X_{i}^{j},{\hat{\Theta }}_{2e}\right)-\mu_{a}\right]}^{2}+Var\left(a_{ij}|X_{i}^{j},{\hat{\Theta }}_{2e}\right)\right\}$$

**M-Step:** ${\hat{\Theta }}_{2\left(e+1\right)}=\arg\max\ln L\left(\Theta_{2}|X_{i}^{j},{\hat{\Theta }}_{2e}\right)$, take the partial derivatives of Equation (20) with respect to $\mu_{a}$, $\sigma_{a}^{2}$, q and equate the results to zero. The results are

(21) $${\left({\hat{\mu }}_{a}\right)}_{e+1}=\frac{1}{\sum_{i=1}^{m}n_{i}}\sum_{i=1}^{m}\sum_{j=1}^{n_{i}}E\left(a_{ij}|X_{i}^{j},{\hat{\Theta }}_{2e}\right)$$

(22) $${\left({\hat{\sigma }}_{a}^{2}\right)}_{e+1}=\frac{1}{\sum_{i=1}^{m}n_{i}}\sum_{i=1}^{m}\sum_{j=1}^{n_{i}}\left\{{\left[E\left(a_{ij}|X_{i}^{j},{\hat{\Theta }}_{2e}\right)-{\left({\hat{\mu }}_{a}\right)}_{e+1}\right]}^{2}+Var\left(a_{ij}|X_{i}^{j},{\hat{\Theta }}_{2e}\right)\right\}$$

(23) $${\hat{q}}_{e+1}=\frac{1}{\sum_{i=1}^{m}n_{i}p_{i}}\sum_{i=1}^{m}\sum_{j=1}^{n_{i}}\sum_{k=1}^{p_{i}}\frac{{\left(\Delta x_{i,k}^{j}-E\left(a_{ij}|X_{i}^{j},{\hat{\Theta }}_{2e}\right)\eta_{i}\left(b\right)\Delta \Lambda \left(t_{i,k}^{j},\theta \right)\right)}^{2}+Var\left(a_{ij}|X_{i}^{j},{\hat{\Theta }}_{2e}\right)\eta_{i}\left(2b\right)\Delta \Lambda^{2}\left(t_{i,k}^{j},\theta \right)}{\eta_{i}\left(b\right)\Delta \tau \left(t_{i,k}^{j},\gamma \right)}$$

Repeat the E-step and M-step iteratively until $\left\|{\hat{\Theta }}_{2(e+1)}-{\hat{\Theta }}_{2e}\right\|<\varepsilon$ $\left(\varepsilon =10^{-6}\right)$.

## 4. Case Studies

### 4.1. Simulation Study

**Figure 1:**
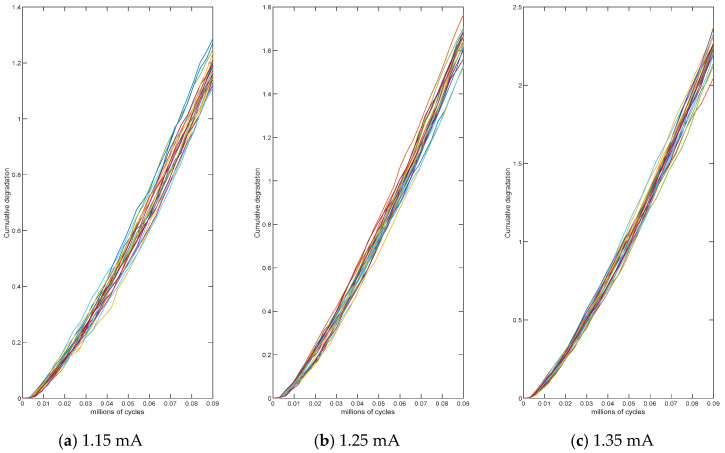
Crack growth accumulation at various stress levels.

A simulation of fatigue crack propagation test data for an alloy product was performed using the Monte Carlo method to validate the proposed methodology. The accelerated stress applied to the alloy product is electrical stress, the normal operational level is $S_{0}=1$ mA. Three accelerated stress levels are considered: $S_{1}=1.15$ mA, $S_{2}=1.25$ mA, $S_{3}=1.35$ mA, the highest allowable stress is $S_{m}=1.35$ mA. The accelerated rate of the degradation process follows a power-law relation, normalized the stresses are $s_{1}=0.4657$, $s_{2}=0.7436$, $s_{3}=1$. Twenty-one samples were measured once every 0.003 million cycles, yielding a total of 30 measurements. The product was considered to have failed when the fatigue crack propagation data reached the failure threshold of D=2.5 inches. The true values of the parameters are set to $\mu_{a}=16$, $\sigma_{a}=1$, q=0.04 and b=1.2. $\Lambda \left(t;\theta \right)=t^{\theta }$ and $\tau \left(t;\gamma \right)=t^{\gamma }$ are two consecutive non-decreasing functions of time t, corresponding to θ=1.3 and γ=1.4, respectively. The detailed simulation results of crack propagation trajectories under different accelerated stress levels are presented in Figure 1.

The validity and advantages of the proposed method are verified using simulated accelerated degradation test data for a specific alloy product. The accelerated degradation model based on the generalized Wiener process proposed is denoted as $M_{1}$. The accelerated degradation model based on the nonlinear Wiener process proposed in [6] is referred to as $M_{2}$. The accelerated degradation model based on the time-scale transformed Wiener process proposed in [5] is denominated as $M_{3}$. $a_{ij}$ is treated as a fixed parameter $a_{ij}=\mu_{a}$, ignoring the intrinsic variations in performance degradation among homogeneous products as $M_{4}$. The estimates of $\left(b,\theta ,\gamma \right)$ can be obtained by maximizing the profile log-likelihood function in Equation (16). We use the MATLAB function “fminsearch” for this optimization. The solution for the parameters is unique and stable across different selected initial values. Subsequently, the estimates of $a_{ij}$ and q can be derived by substituting the estimates of $\left(b,\theta ,\gamma \right)$ into Equations (14) and (15). Furthermore, these estimates of the parameter can serve as the initial values for the EM algorithm. For the calculation of ${\left({\hat{\mu }}_{a}\right)}_{e+1}$, ${\left({\hat{\sigma }}_{a}^{2}\right)}_{e+1}$, ${\hat{q}}_{e+1}$, the E-step and M-step are repeated iteratively until $\left\|{\hat{\Theta }}_{2(e+1)}-{\hat{\Theta }}_{2e}\right\|<\varepsilon$ $\left(\varepsilon =10^{-6}\right)$. For comparative analysis, the parameter estimation results obtained using the MLE and EM algorithms are summarized in Table 1.

To assess model fitting performance, the AIC is adopted as the evaluation metric. The AIC is defined as $AIC=2p-2\ln L\left(\Theta \right)$, where p denotes the number of parameters in the parameter set Θ. A smaller AIC value indicates a better model fit. The calculated AIC values for models $M_{1}$, $M_{2}$, $M_{3}$ and $M_{4}$ indicate that $M_{1}$ yields the highest log-likelihood function value and the smallest AIC value, suggesting that model $M_{1}$ provides the best fit and is more appropriate for describing the degradation process. Considering the complexity of actual degradation, the accelerated degradation model incorporating random effects provides a more realistic description of practical scenarios. Under the normal stress, the product’s mean time to failure $MTTF_{j}=E\left(T|s_{0}\right)=\int_{0}^{\infty }tf_{j}\left(t|s_{0}\right)dt$, j=1,2,3,4 and its 95% confidence interval (CI) results for the product are shown in Table 2. The true mean time to failure (MTTF) is 0.2413 million cycles. The MTTF estimated by model $M_{1}$ is 0.2388 million cycles, which is relatively close to the true value. Models $M_{2}$ and $M_{3}$ underestimate the product’s MTTF, whereas model $M_{4}$ slightly overestimates it.

**Figure 2:**
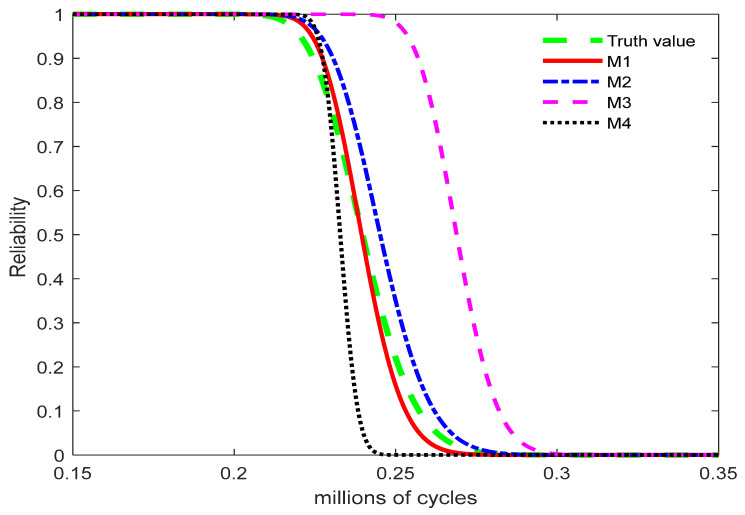
Reliability curve of the model.

Figure 2 presents the reliability function curves obtained under the normal stress level of $S_{0}=1$ mA, including those corresponding to the true value and Models $M_{1}$, $M_{2}$, $M_{3}$, and $M_{4}$. Model $M_{3}$ tends to overestimate product reliability, whereas model $M_{4}$ yields lower reliability estimates than the other models, indicating a tendency to underestimate reliability. The reliability curve obtained from model $M_{1}$ is closer to the true reliability curve than that from model $M_{2}$.

**Figure 3:**
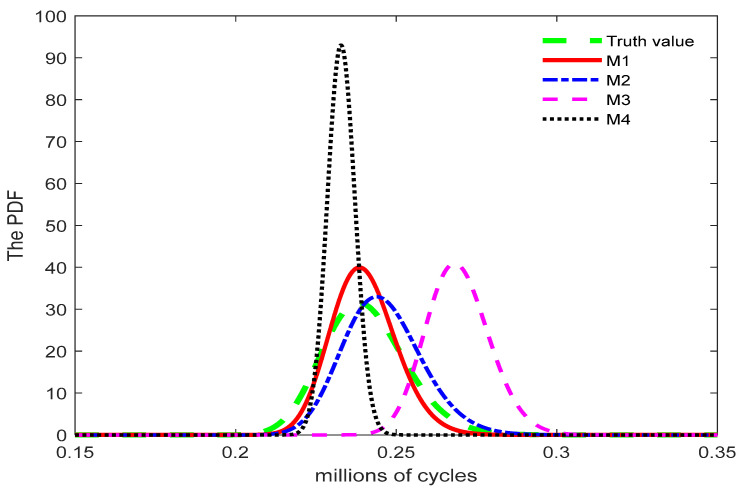
Probability density curve of the model.

Figure 3 presents the PDFs of the failure time corresponding to the true value and models $M_{1}$, $M_{2}$, $M_{3}$ and $M_{4}$. The 95% CI of the failure time predicted by model $M_{1}$ encompasses the true failure time of the product. Furthermore, the 95% confidence interval predicted by Model $M_{1}$ is closer to the interval constructed using the true value, indicating its higher prediction accuracy. Consequently, the evaluation results demonstrate that the proposed Model $M_{1}$ provides a reliable fit to the simulation data. However, the CI predicted by model $M_{4}$ is overly narrow and fails to include the true lifespan, this means that the fitting results of the model without considering random effects are worse than those of the other three models.

### 4.2. Application to the Stress Relaxation Data

**Figure 4:**
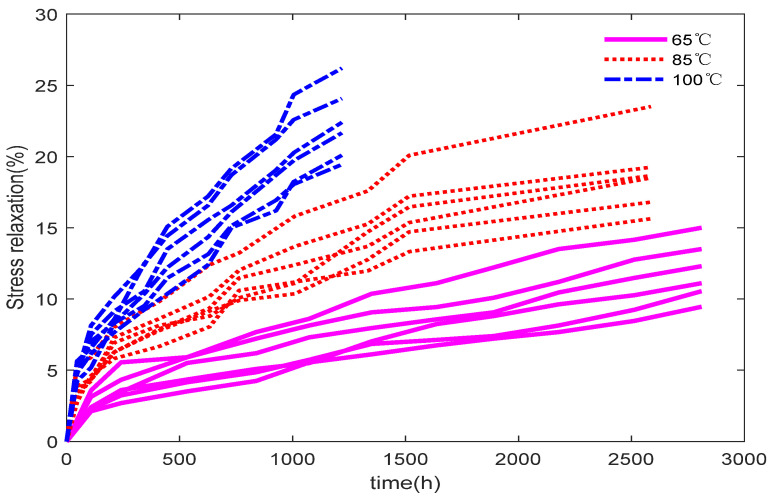
Degradation trajectories of stress relaxation data under accelerated stress conditions.

Stress relaxation refers to the loss of elasticity in a product under constant stress. If the initial stress in a product component is $V_{0}$ and it decreases to ΔV after a certain period, then $\Delta V/V_{0}$ is defined as the stress relaxation amount. For example, electrical connectors such as patch panels may fail due to stress relaxation. If stress relaxation exceeds 30% under constant stress, the failure threshold is defined as D=30%. Take the stress relaxation data from the literature [30] (Example 8.7, pp. 351) as an example, and the Arrhenius model is adopted as the acceleration model. The temperature stress levels are 65 °C, 85 °C,100 °C, with 6 samples per stress level. Degradation data is collected at various time points. The ADT data and their corresponding measurement times are presented in Appendix A (Table A1 and Table A2). The constant stress level is $S_{0}=40$ °C, the maximum temperature is $S_{3}=100$ °C, and the normalized stresses are $s_{1}=0.4598$, $s_{2}=0.7814$, $s_{3}=1$. The imputed value for the missing data is 7.12. The degradation trajectories of the samples under the three temperature stress levels are shown in Figure 4. It can be observed that these trajectories exhibit nonlinear patterns, indicating that a nonlinear Wiener process is appropriate for modeling the stress relaxation data. Moreover, noticeable individual differences exist among the samples at each temperature level, highlighting the necessity of accounting for product-to-product heterogeneity in the modeling process.

The initial values θ,γ,b are set to (1, 1, 1), and the fminsearch function is used for iterative optimization. The EM algorithm is employed to estimate the parameters $\mu_{a}$, $\sigma_{a}^{2}$ and η, the resulting parameter estimates are summarized in Table 3. The proposed accelerated degradation model based on the generalized Wiener process is denoted as $M_{1}$. The nonlinear Wiener process model from the literature [8], in which γ=1, is denoted as $M_{2}$. Additionally, the Wiener process model with time-scale transformation from the literature [7], where θ=γ is referred to as $M_{3}$. An alternative accelerated degradation model based on the generalized Wiener process is denoted as $M_{4}$, which $a_{ij}$ is taken as a fixed parameter, $a_{ij}=\mu_{a}$, and the variability in performance degradation among similar products is ignored.

To evaluate the goodness of fit of different models, the AIC is adopted as the model selection criterion. By calculating the AIC values of models $M_{1}$, $M_{2}$, $M_{3}$, $M_{4}$, it is observed that model $M_{1}$ achieves the highest log-likelihood function value $\ln L\left(\Theta \right)$ and the lowest AIC value. This suggests that model $M_{1}$ provides the best fit and is more suitable for describing the degradation process of stress relaxation in the patch panels. Considering the complexity of the actual degradation process, the accelerated degradation model $M_{1}$, which incorporates random effects, exhibits better fitting performance compared to the fixed-effects accelerated model $M_{4}$. The MTTFs and 95% CIs are summarized in Table 4.

**Figure 5:**
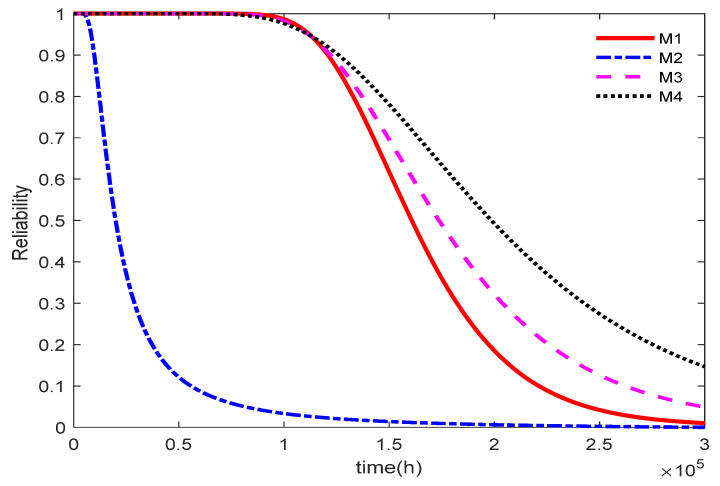
Reliability curves of product life under different models.

**Figure 6:**
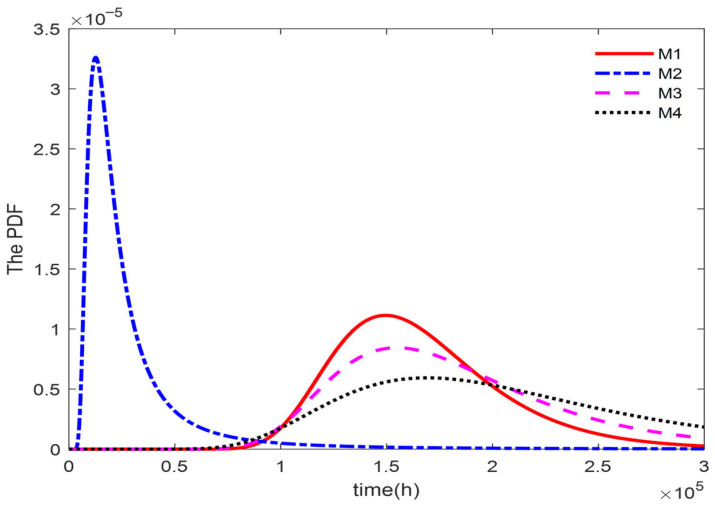
Probability density curves of product life under different models.

**Figure 7:**
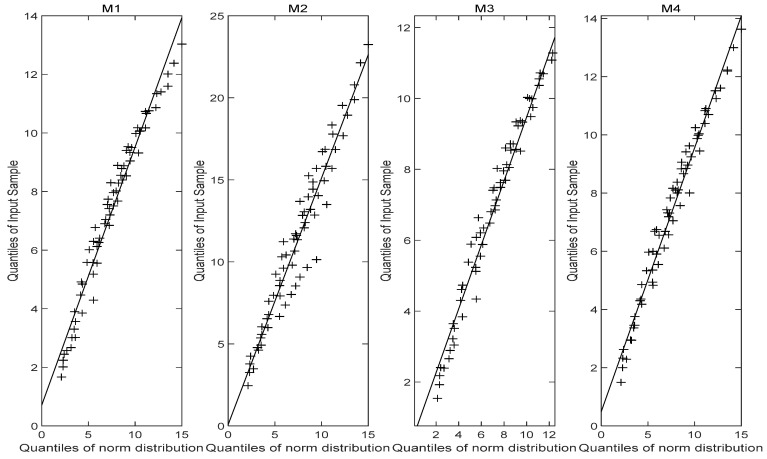
Q-Q plot (65 °C).

**Figure 8:**
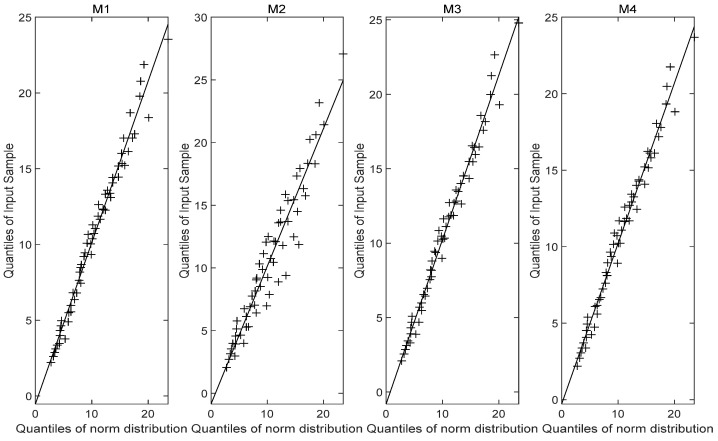
Q-Q plot (85 °C).

**Figure 9:**
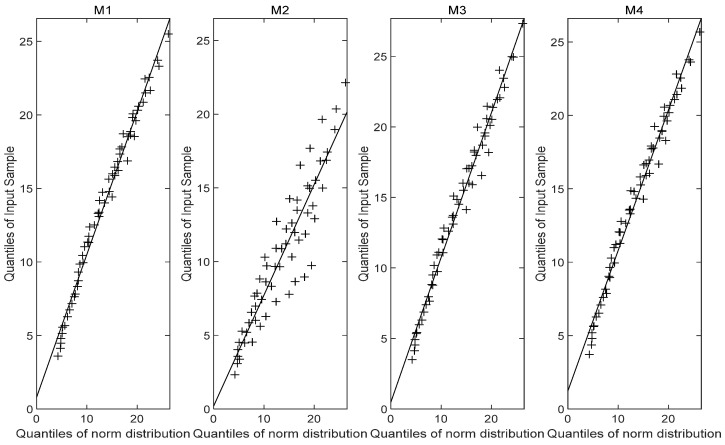
Q-Q plot (100 °C).

Figure 5 illustrates the reliability curves of models $M_{1}$, $M_{2}$, $M_{3}$ and $M_{4}$ under normal use stress $S_{0}=40$ °C, while Figure 6 displays the corresponding probability density curves. From the graphical analysis, the PDFs of the failure time for models $M_{1}$ and $M_{3}$ are very similar, with both exhibiting comparable distribution characteristics. In contrast, the results from model $M_{2}$ show a noticeable left-skewed trend, indicating a shorter predicted lifespan, and diverge significantly from those of models $M_{1}$, $M_{3}$ and $M_{4}$, suggesting that model $M_{2}$ is unreliable. A plausible explanation is that the nonlinear Wiener-process-based model may not be suitable for the stress-relaxation degradation data. The MTTF predicted by model $M_{1}$ is more consistent with the MTTFs predicted by models $M_{3}$ and $M_{4}$, while differing significantly from that of model $M_{2}$. Furthermore, the probability density function produced by model $M_{1}$ is more concentrated, indicating higher predictive accuracy. The fundamental concept of a Q–Q plot is to graph the quantiles of the empirical distribution against the theoretical quantiles of a specified model in the Cartesian coordinate system, based on observed sample data. If the points approximately form a straight line, it suggests a good fit between the observations and the distribution model. The Q-Q plot Figure 7, Figure 8 and Figure 9 comparison also reveals that the quantiles of model $M_{1}$ exhibit strong linearity with respect to the test data, indicating a better fit than the other three models across the three stress levels. These visual results are consistent with the AIC, confirming the accuracy of the established model.

## 5. Conclusions

During the accelerated degradation test under constant stress, an accelerated degradation model is developed in which both the drift and diffusion coefficients are modeled as functions of the accelerating stress. To account for individual differences among products, a normally distributed random effect is incorporated into the model. The parameters are estimated using the MLE method in conjunction with the EM algorithm. Model comparison and reliability analysis are conducted. The effectiveness and practical applicability of the proposed method are validated through a numerical simulated example and an application to the stress relaxation data. The results indicate that the accelerated degradation model with random effects performs better than the corresponding model with fixed effects. Among the models evaluated, the proposed accelerated degradation model based on the generalized Wiener process demonstrates the highest estimation efficiency. Nonetheless, several issues remain open for future investigation. This study considers randomness only in the drift coefficient. However, randomness in the diffusion coefficient may also play a significant role and should be further investigated. Additionally, the current research focuses primarily on single-stress accelerated degradation models. Future studies should focus on multi-stress accelerated degradation scenarios, as they more accurately represent actual operating environments and pose complex challenges that require comprehensive analysis and further methodological development.

## Figures and Tables

**Table 1:** The parameters of four degradation models with the CSADT simulated degradation data.

	$\mu_{a}$	$\sigma_{a}$	b	θ	γ	q
Truth value	16	1	1.2	1.3	1.4	0.04
$M_{1}$	17.7006	0.9677	1.0901	1.3695	1.0645	0.0086
$M_{2}$	17.3076	1.0678	1.0643	1.3642	1	0.0085
$M_{3}$	15.4473	0.6883	1.2732	1.3868	1.3868	0.0216
$M_{4}$	18.4373	−	1.1944	1.3712	1.0821	0.0193

**Table 2:** Model evaluation results.

	$\ln L\left(\Theta \right)$	AIC	MTTF	CI
Truth value	−	−	0.2413	[0.2172, 0.2677]
$M_{1}$	7.3774 × 10^3^	−1.4743 × 10^4^	0.2388	[0.2214, 0.2611]
$M_{2}$	7.1753 × 10^3^	−1.4341 × 10^4^	0.2449	[0.2237, 0.2720]
$M_{3}$	7.4358 × 10^3^	−1.4474 × 10^4^	0.2693	[0.2510, 0.2896]
$M_{4}$	6.7516 × 10^3^	−1.3588 × 10^4^	0.2329	[0.2245, 0.2414]

**Table 3:** Parameters of the accelerated degradation model.

	$\mu_{a}$	$\sigma_{a}$	b	θ	γ	q
$M_{1}$	0.0999	0.0096	2.0150	0.4758	0.5006	0.0071
$M_{2}$	0.3942	0.1091	0.5918	0.4374	1	3.0256 × 10^−4^
$M_{3}$	0.0925	0.0121	2.1012	0.4791	0.4791	0.0083
$M_{4}$	0.1179	−	2.0133	0.4525	0.6474	0.0096

**Table 4:** Results of model evaluation.

	$\ln L\left(\Theta \right)$	AIC	MTTF	CI
$M_{1}$	−60.1128	132.2256	1.6372 × 10^5^	[104,937.8751, 256,149.6332]
$M_{2}$	−113.0995	236.1990	2.1228 × 10^4^	[7204.4619, 115,221.4058]
$M_{3}$	−61.7570	133.5140	1.6794 × 10^5^	[102,645.0685, 278,910.1876]
$M_{4}$	−181.5293	373.0586	1.6119 × 10^5^	[98,371.9130, 289,176.0514]

## Data Availability

Data are contained within the article.

## Abbreviations

The following abbreviations are used in this manuscript:

ADT	Accelerated degradation test
CSADT	Constant-stress accelerated degradation test
MLE	Maximum likelihood estimation
EM	Expectation maximization
FHT	First hitting time
PDF	Probability density function
AIC	Akaike information criterion
MTTF	Mean time to failure
CI	Confidence interval

## Appendix A

The stress relaxation data and measurement times are provided in Table A1 and Table A2. The symbol ‘*’ denotes instances where no data was recorded at a given time.

**Table A1:** Stress relaxation degradation data under different temperatures.

T	ID	Stress Relaxation										
65 °C	1	2.12	2.70	3.52	4.25	5.55	6.12	6.75	7.22	7.68	8.46	9.46
	2	2.29	3.24	4.16	4.86	5.74	6.85	*	7.40	8.14	9.25	10.55
	3	2.40	3.61	4.35	5.09	5.50	7.03	8.24	8.81	9.629	10.27	11.11
	4	2.31	3.48	5.51	6.20	7.31	7.96	8.57	9.07	10.46	11.48	12.31
	5	3.14	4.33	5.92	7.22	8.14	9.07	9.44	10.09	11.20	12.77	13.51
	6	3.59	5.55	5.92	7.68	8.61	10.37	11.11	12.22	13.51	14.16	15.00
85 °C	7	2.77	4.62	5.83	6.66	8.05	10.61	11.20	11.98	13.33	15.64	
	8	3.88	4.37	6.29	7.77	9.16	9.90	10.37	12.77	14.72	16.80	
	9	3.18	4.53	6.94	8.14	8.79	10.09	11.11	14.72	16.47	18.66	
	10	3.61	4.37	6.29	7.87	9.35	11.48	12.40	13.70	15.37	18.51	
	11	3.42	4.25	7.31	8.61	10.18	12.03	13.70	15.27	17.22	19.25	
	12	5.27	5.92	8.05	9.81	12.40	13.24	15.83	17.59	20.09	23.51	
100 °C	13	4.25	5.18	8.33	9.53	11.48	13.14	15.55	16.94	18.05	19.44	
	14	4.81	6.16	7.68	9.25	10.37	12.40	15.00	16.20	18.24	20.09	
	15	5.09	7.03	8.33	10.37	12.22	14.35	16.11	18.70	19.72	21.66	
	16	4.81	7.50	9.16	10.55	13.51	15.55	16.57	19.07	20.27	22.40	
	17	5.64	6.57	8.61	12.50	14.44	16.57	18.70	21.20	22.59	24.07	
	18	4.72	8.14	10.18	12.40	15.09	17.22	19.16	21.57	24.35	26.20	

* indicates missing data.

**Table A2:** Inspection time under different temperatures.

T	Measurement Time Epochs (in Hours)										
65 °C	108	241	534	839	1074	1350	1637	1890	2178	2513	2810
85 °C	46	108	212	408	632	764	1011	1333	1517	2586	
100 °C	46	108	212	344	446	626	729	927	1005	1218	
